# Changes in Waist Circumference and the Incidence of Diabetes in Middle-Aged Men and Women

**DOI:** 10.1371/journal.pone.0023104

**Published:** 2011-08-04

**Authors:** Tina Landsvig Berentzen, Marianne Uhre Jakobsen, Jytte Halkjaer, Anne Tjønneland, Thorkild I. A. Sørensen, Kim Overvad

**Affiliations:** 1 Institute of Preventive Medicine, Copenhagen University Hospital, Copenhagen, Denmark; 2 Department of Epidemiology, School of Public Health, Aarhus University, Aarhus, Denmark; 3 The Danish Cancer Society, Institute of Cancer Epidemiology, Copenhagen, Denmark; 4 Department of Cardiology, Center for Cardiovascular Research, Aalborg Hospital, Aarhus University Hospital, Aalborg, Denmark; Karolinska Insitutet, Sweden

## Abstract

**Background:**

Waist circumference (WC) is positively associated with diabetes, but the association with changes in WC (DWC) is less clear. We investigated the association between DWC and the subsequent risk of diabetes in middle-aged men and women, and evaluated the influence from concurrent changes in body mass index (DBMI).

**Methodology/Principal Findings:**

Data on 15,577 men and 20,066 women from the Danish Diet, Cancer and Health study were analyzed. Anthropometry was assessed in 1993–97 and 1999–02. Information on diabetes was obtained from The Danish National Diabetes Register. Hazard ratios (HR) were calculated from Cox' proportional hazard models with individuals considered at risk from 1999–02 until December 31 2006. During 5.4 years of follow-up, 1,027 and 876 new cases of diabetes occurred among men and women, respectively. WC was positively associated with diabetes in both sexes also with adjustment for covariates and BMI. DWC was positively associated with diabetes in women, but not in men (HR per 5 cm change = 1.09 (1.04∶1.15) in women, and 1.00 (0.94, 1.07) in men with adjustment for covariates, baseline WC, BMI and DBMI). Associations with DWC were not notably different in sub-groups stratified according to baseline WC or DBMI, or when individuals with diseases or diabetes occurring within the first years of follow-up were excluded.

**Conclusions/Significance:**

While this study confirmed that WC is positively associated with the risk of diabetes in middle-aged men and women, it surprisingly showed that changes in WC were not associated with the subsequent risk of diabetes in men, and only weakly positively associated with the risk of diabetes in women. Accordingly, these findings suggest that a reduction in WC may be a weak or insufficient or target for prevention of diabetes in middle-aged men and women.

## Introduction

Obesity is a strong risk factor for diabetes [1]. Weight gain increases the risk of diabetes, and even small reductions in body weight reduce the risk of diabetes [1]. Accordingly, weight management has a crucial role in the prevention and treatment of diabetes [1].

Individuals differ in their regional distribution of body fat. Anthropometric measures of abdominal fatness (e.g. waist circumference (WC)) appears to be more strongly associated with the risk of diabetes than anthropometric measures of general fatness (e.g. body mass index (BMI)) [2]–[6]. This has predominantly been attributed to accumulation of intra-abdominal fat, which is strongly associated with metabolic complications and possibly also with development of diabetes [7]–[9]. In contrast, anthropometric measures of peripheral fatness are inversely associated with the risk of diabetes [10] possibly due to favorable metabolic effects of the skeletal muscles and the peripheral body fat [11]. Although men accumulate more abdominal fat than women, abdominal fatness seems more strongly associated with diabetes in women than men [8], [9].

Several studies have shown that WC measured at one point in time is strongly associated with the risk of diabetes, but the association with changes in WC is less clear. Waist changes were positively associated with development of diabetes in young adults in the CARDIA study [12], but estimates for each sex or adjusted for overall weight changes were not shown. Waist changes were also associated with development of diabetes in men in the Health Professionals Follow-up Study [13], but only major waist gain was associated with diabetes, and the association was weak after adjustment for overall weight change [13]. None of these studies considered women separately, and it is not clear if waist changes are associated with development of diabetes beyond and throughout the range of the overall weight changes.

We investigated the association between changes in WC and the subsequent risk of diabetes in middle-aged men and women, and evaluated whether the association was influenced by concurrent changes in BMI.

## Materials and Methods

In 1993–97, a random sample of 160,725 individuals aged 50–64 years were invited to the Danish prospective study ‘Diet, Cancer and Health’. A total of 57,053 accepted the invitation (569 were later excluded due to a cancer diagnosis, which was not, due to processing delays, registered in the Danish Cancer Register at the time of the invitation). Participants filled in questionnaires and were clinically examined. In 1999–2002, repeated information was collected with questionnaires. The Danish Data protection Agency and the regional Ethical Committees in Copenhagen and Aarhus approved the study, which was in accordance with the Helsinki Declaration II. Participants signed a written consent before participating. Details of the study are described elsewhere [14].

### Exposures and covariates

In 1993–97, technicians measured the individuals' height (nearest 0.5 cm without shoes) and weight (nearest 0.1 kg using a digital scale, with light clothes/underwear). The WC was measured (nearest 0.5 cm) with a measuring tape at the smallest horizontal circumference between the ribs and iliac crest (natural waist), or, in case of an indeterminable WC narrowing, halfway between the lower rib and the iliac crest. In 1999–02, individuals received a self-administrated questionnaire and reported their weight (kg) and WC (cm) measured at the level of the umbilicus using an enclosed paper measuring tape. BMI (kg/m^2^) was calculated as weight per height squared. Change in WC (DWC) (cm) and change in BMI (DBMI) (kg/m^2^) was calculated as the value in 1993–97 subtracted from the value in 1999–02.

Covariates, assessed with the 1999–02 questionnaire, were used: *smoking habits* (never, ex, current smoker of <15 g/day, 15–25 g/day, >25 g/day), *sports/vigorous activity* (0 versus>0 hours/wk) [15], [16], *total energy intake* (including alcohol) (KJ/day) [15], [17], diet quality assessed as a modified *Mediterranean diet score* [18], *drinking pattern* (abstainer, 0–3 times/month, 1–4 times/wk, 5–6 times/wk, daily), *educational level* (length of education: <8 years (basic school), 8–10 years (vocational education, higher education of 1–2 years), >10 years (vocational education, higher education of more than 2 years)) [14], and in women *menopausal status* (pre, post, unknown). Moreover, we identified individuals with diagnosed chronic diseases *(yes/no)* (defined according to a previously developed classification) [19] occurring before examination in 1999–02 by linkage to the National Hospital Discharge Register that includes all hospitalisations since 1970 [20].

### Outcome

Information on diabetes was obtained by linkage to the Danish National Diabetes Register [21], [22] which include individuals that have a diagnosis of diabetes registered in The National Hospital Discharge Register (International Classification of Disease (ICD) 8: codes 249–250 and ICD10 codes DE10–DE14, DH36.0, DO24 (excluding DO24.4), were registered in The National Health Insurance Service Register with chiropody for patients with diabetes, a fifth blood-glucose measurement within one year, two blood glucose measurements per year in five consecutive years, or were registered in The Drug Prescription Register with purchase of oral glucose lowering drugs within 6 months or prescribed insulin.

### Statistical Analyses

**Figure 1:**
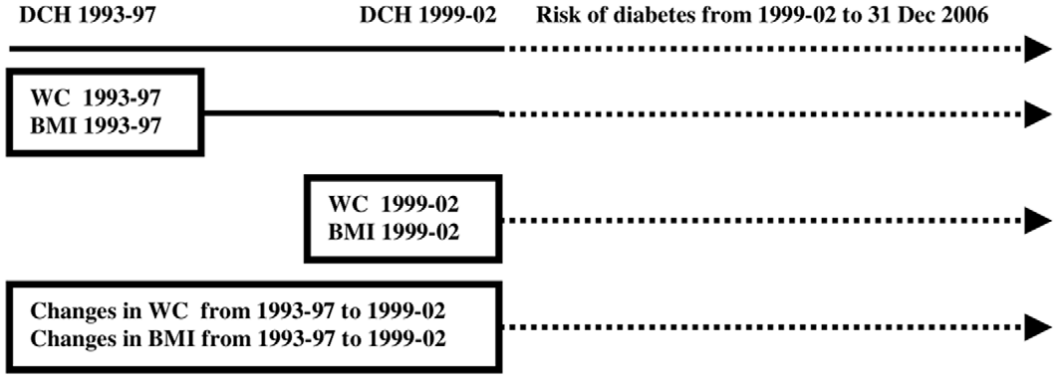
Body Mass Index and Waist Circumference and Changes in Body Mass Index and Waist Circumference in relation to the Subsequent Risk of Diabetes.

Hazard ratios (HR) of diabetes were calculated from Cox proportional hazard models with years since the examination in 1999–02 as time axis, so that individuals were considered at risk from 1999–02 until time at diabetes, death, emigration/disappearance or December 31 2006, whichever came first (figure 1). Analyses were conducted for each sex separately. Sex differences were formally tested on the multiplicative scale by cross-product terms using Wald tests.

Analyzing continuous exposures in epidemiology has been widely debated [23]. We used restricted cubic splines as these provide smooth curves that could be a plausible biological appearance for the investigated associations [23]. WC in 1993–97 was included as restricted cubic splines (3 knots) [24] in models with age in 1999–02, years between examinations and presence of chronic disease. Covariates were added in a second step, and BMI in 1993–97 was added in a third step. Similar analyses were conducted for BMI in 1993–97 with WC in 1993–97 added in the third step, and for WC and BMI measured in 1999–02. The DWC was included as restricted cubic splines (3 knots) [24] in models with age in 1999–02, years between examinations, presence of chronic disease and WC in 1993–97. Covariates were added in a second step, and DBMI and BMI in 1993–97 were added in a third step. Similar analyses were conducted for DBMI with WC in 1993–97 and DWC added in the third step. Continuous covariates were included as restricted cubic splines (3 knots) [24]. Splines were plotted to visually assess the shape of the associations, and associations were formally tested by Wald tests. BMI, WC, DBMI and DWC were also included as continuous linear variables in models with covariates added in the three steps described above. These results are presented when linearity of these associations were accepted.

The proportional hazard assumption was assessed with a log-rank test based on Schoenfeld residuals. No violations were detected in women, but there was indication of violation due to age in 1999–02 in men. Models were therefore stratified by age in 1999–02, so the baseline hazard was estimated separately within four age-strata (quartiles). Associations between the anthropometric variables and diabetes were similar in these and the models with age included as splines. No violation of the proportional hazard assumption was detected in the models with age in 1999–02 in strata.

#### Subgroups analyses

To explore if the association between DWC and diabetes was equal throughout the range of the DBMI, the association between DWC and diabetes was investigated in groups with loss (DBMI< = 0) and gain in BMI (DBMI>0). Similarly, the association between DWC and diabetes was investigated in groups with a high and low WC in 1993–97 (cut-off at the mean WC (95 cm in men and 81 cm in women)).

Diabetes may go undiagnosed for years [25] and may, as other chronic diseases, induce changes in anthropometry [26], which implies risks of bias due to reverse causation. We explored this by exclusion of individuals with diagnosed chronic diseases [19] occurring before follow-up in 1999–02 and diabetes cases occurring in the first one to five years of follow-up.

Analyses were conducted in STATA version 9.2 (Stata Corporation, College Station, Texas; www.stata.com). Statistically significant differences were defined as differences with p<0.05.

## Results

Between the examinations in 1993–97 and 1999–02, 1778 individuals died and 460 emigrated/disappeared leaving 54,246 eligible for re-invitation. Among these, 5,865 did not respond, 2,858 did not want to participate, 649 had questionnaires with errors, and for 1,051 information on follow-up time, anthropometry or covariates was missing leaving 20,667 men and 23,156 women. Of these, 1,245 men and 878 women were excluded due to diabetes before follow-up in 1999–02, and 4,057 were excluded due to extreme values on the anthropometric variables (values below the 0.5 and above the 99.5 sex-specific percentiles of WC and BMI, and below the 2.5 and above the 97.5 sex-specific percentiles DWC and DBMI). Thus, 17,577 men and 20,066 women were eligible for the current study.

Table 1 provides the basic description of the cohort. The median observation time from the examination in 1999–02 to diabetes or censoring was 5.4 years in both sexes. In this period, 1,027 and 876 new cases of diabetes occurred among men and women, respectively. The median WC was 94 cm in men and 79 cm in women in 1993–97. During the 5.3 years between the two examinations, the median increase in WC was 3 cm in men and 7 cm in women. In men, 5,544 (32%) had a loss in WC and 12,033 (68%) had a gain in WC. In women, 3,045 (15%) had a loss in WC and 17,021 (85%) had a gain in WC. The Pearson correlation between WC and BMI in 1993–97 was high in both sexes (0.84), but modest between DWC and DBMI in men (0.42) and women (0.36).

**Table 1:** Characteristics of Men and Women from the Danish Diet, Cancer and Health Study included in the Present Study.

	Men (n = 17,577)	Women (n = 20,066)
	Median (5–95%tile)	Median (5–95%tile)
Age (year) in 1993–97	55.9 (50.7∶64.2)	56.1 (50.8∶64.1)
Age (year) in 1999–02	61.3 (56.0∶69.5)	61.4 (56.0∶69.5)
Years btw examinations in 1993–97 and in 1999–02	5.3 (5.0∶5.8)	5.3 (5.0∶5.9)
Years btw examination in 1999–02 and end of follow-up	5.4 (4.4∶6.5)	5.4 (4.5∶6.5)
BMI (kg/m^2^) in 1993–97	25.9 (21.7∶31.6)	24.5 (20.0∶32.1)
BMI (kg/m^2^) in 1999–02	25.8 (21.7∶31.6)	24.4 (19.9∶32.1)
WC (cm) in 1993–97	94 (82∶110)	79 (67∶99)
WC (cm) in 1999–02	97 (85∶112)	87 (72∶108)
Changes in BMI (kg/m^2^) btw 1993–97 and 1999–02	0.0 (−1.9∶1.8)	−0.1 (−2.3∶2.1)
Changes in WC (cm) btw 1993–97 and 1999–02	3 (−6∶11)	7 (−3∶20)
Mediterranean Diet Score	5 (3, 8)	5 (3, 7)
Energy Intake (Mj/d)	10.2 (6.5, 15.3)	8.1 (5.2, 12.6)
Chronic diseased	28%	28%
Current smokers	30%	25%
Physically inactive	42%	36%
Daily alcohol intake	33%	19%
Less than 8 years of school	32%	29%
Postmenopausal	-	79%

### Single measurements of BMI and WC

**Figure 2:**
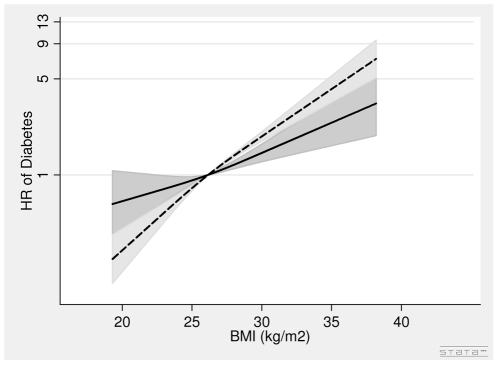
Hazard Ratios and 95% Confidence Intervals of Diabetes according to Body Mass Index in 1993–97 without (dashed line) and with adjustment (solid line) for Waist Circumference in Men from the Danish Diet, Cancer and Health Study. Abbreviations: BMI, body mass index. HR, hazard ratio. Lines are the hazard ratios (areas the 95%-confidence intervals) derived from Cox's proportional-hazard models where BMI was included as restricted cubic splines (3 knots). Reference points are the mean BMI. Years since the examination in 1999–02 was used as time axis. *Adjustments*: years between examinations, age in 1999–02, chronic diseases, smoking habits, Mediterranean diet score, energy intake, education, drinking pattern, sports activity. BMI in 1993–97: Test of linearity *P* = 0.24 (linear). Test of effect *P*<0.0001 (with adjustment for covariates, but without adjustment for waist circumference). BMI in 1993–97: Test of linearity *P* = 0.58 (linear). Test of effect *P*<0.0001 (with adjustment for covariates and waist circumference).

**Figure 3:**
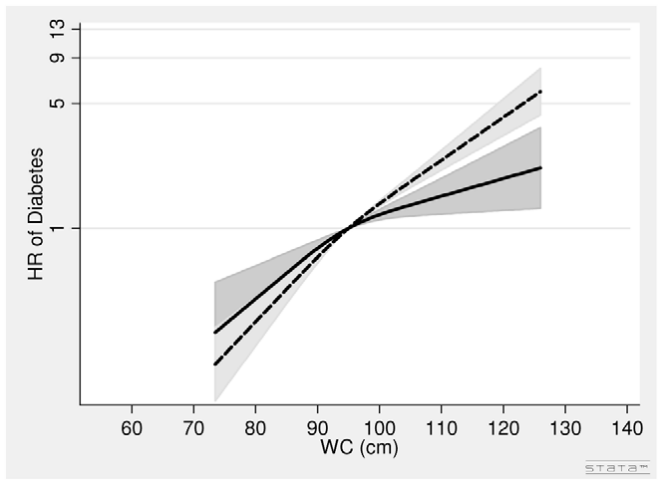
Hazard Ratios and 95% Confidence Intervals of Diabetes according to Waist Circumference in 1993–97 without (dashed line) and with (solid line) adjustment for Body Mass index in Men from the Danish Diet, Cancer and Health Study. Abbreviations: HR, hazard ratio. WC, waist circumference. Lines are the hazard ratios (areas the 95%-confidence intervals) derived from Cox's proportional-hazard models where WC was included as restricted cubic splines (3 knots). Reference points are the mean WC. Years since the examination in 1999–02 was used as time axis. *Adjustments*: years between examinations, age in 1999–02, chronic diseases, smoking habits, Mediterranean diet score, energy intake, education, drinking pattern, sports activity. WC in 1993–97: Test of linearity *P* = 0.05 (linear). Test of effect *P*<0.0001 (with adjustment for covariates, but without adjustment for body mass index). WC in 1993–97: Test of linearity *P* = 0.08 (linear). Test of effect *P*<0.0001 (with adjustment for covariates and body mass index).

In men, the associations between BMI in 1993–97, WC in 1993–97 and diabetes were positive (table 2, figure 2). A one unit higher BMI was associated with a 10% (HR = 1.10 (1.06∶1.14) per 1 kg/m^2^) higher risk of diabetes after adjusting for covariates and WC. A 5 cm larger WC was associated with a 19% (HR = 1.19 (1.12∶1.27) per 5 cm) higher risk of diabetes after adjusting for covariates and BMI (table 2, figure 3).

**Table 2:** Hazard Ratios and 95% Confidence Intervals of Diabetes according to Body Mass Index and Waist Circumference in 1993–97 and 1999–97 in Men and Women from the Danish Diet, Cancer and Health Study.

	Crude	Adjusted	Adjusted+WC	Adjusted+BMI
Men	HR (95% CI)^c^	HR (95% CI)^c^^,^ d	HR (95% CI)^c^^,^ d ^,^ e	HR (95% CI)^c^^,^ d ^,^ e
BMI in 1993–97 (kg/m^2^)	1.19 (1.17, 1.21)	1.19 (1.17, 1.21)	1.10 (1.06, 1.14)^a^	-
BMI in 1999–02 (kg/m^2^)	1.18 (1.16, 1.20)	1.18 (1.16, 1.20)	1.14 (1.11, 1.17)^a^	-
WC in 1993–97 (5 cm)	1.38 (1.33, 1.42)	1.37 (1.32, 1.41)	-	1.19 (1.12, 1.27)^b^
WC in 1999–02 (5 cm)	1.32 (1.28, 1.37)	1.31 (1.27, 1.36)	-	1.09 (1.04, 1.15)^b^
**Women**				
BMI in 1993–97 (kg/m^2^)	1.15 (1.13, 1.16)	1.15 (1.13, 1.16)	1.02 (0.99, 1.05)^a^	-
BMI in 1999–02 (kg/m^2^)	1.16 (1.14, 1.17)	1.15 (1.14, 1.17)^f^	1.08 (1.05, 1.10)^a^	-
WC in 1993–97 (5 cm)	1.36 (1.32, 1.40)	1.35 (1.31, 1.39)	-	1.31 (1.23, 1.38)^b^
WC in 1999–02 (5 cm)	1.31 (1.28, 1.35)	1.30 (1.27, 1.33)	-	1.17 (1.12, 1.23)^b^^,^ f

Abbreviations: BMI, body mass index. CI, confidence interval. HR, hazard ratio. WC, waist circumference.

a The association was different in men and women (interaction, *P*<0.05).

b The association was not different in men and women (interaction, *P>0.05*).

c Adjusted for years between examinations, age in 1999–02 and chronic diseases.

d Adjusted for smoking habits, Mediterranean diet score, energy intake, education, drinking pattern, sports activity and menopausal status.

e WC added to analyses of BMI, and BMI added to analyses of WC.

f Associations were accepted to be linear, except for small deviations for BMI and WC in 1999–02 in women , see figure S1.

**Figure 4:**
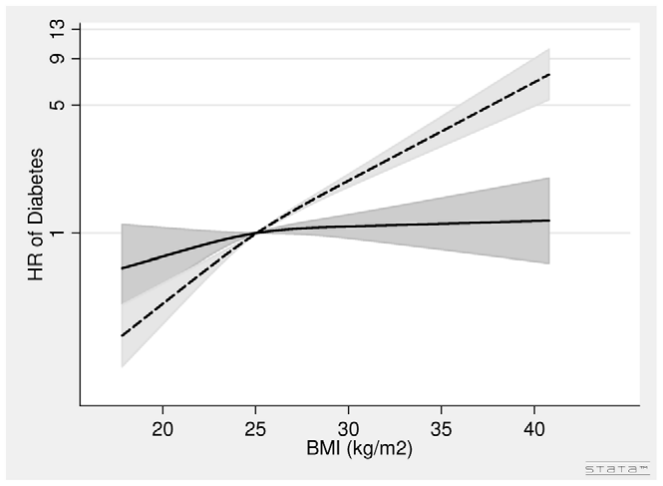
Hazard Ratios and 95% Confidence Intervals of Diabetes according to Body Mass Index in 1993–97 without (dashed line) and with (solid line) adjustment for Waist Circumference in Women from the Danish Diet, Cancer and Health Study. Abbreviations: BMI, body mass index. HR, hazard ratio. Lines are the hazard ratios (areas the 95%-confidence intervals) derived from Cox's proportional-hazard models where BMI was included as restricted cubic splines (3 knots). Reference points are the mean BMI. Years since the examination in 1999–02 was used as time axis. *Adjustments*: years between examinations, age in 1999–02, chronic diseases, smoking habits, Mediterranean diet score, energy intake, education, drinking pattern, sports activity, menopausal status. BMI in 1993–97: Test of linearity *P* = 0.12 (linear). Test of effect *P*<0.0001 (with adjustment for covariates, but without adjustment for waist circumference). BMI in 1993–97: Test of linearity *P* = 0.25 (linear). Test of effect *P* = 0.22 (with adjustment for covariates and waist circumference).

**Figure 5:**
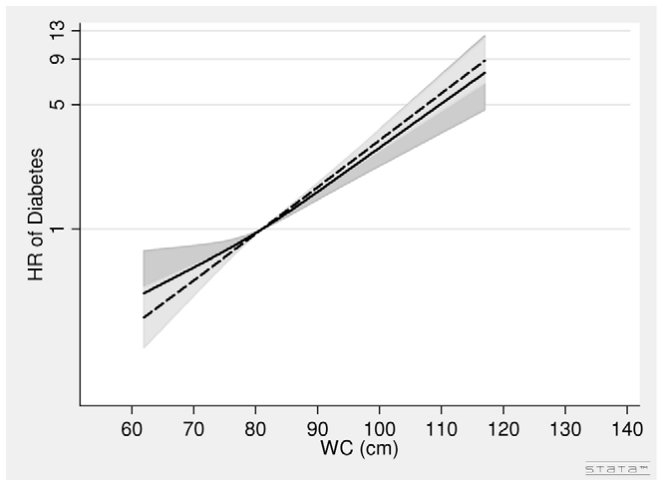
Hazard Ratios and 95% Confidence Intervals of Diabetes according to Waist Circumference in 1993–97 without (dashed line) and with (solid line) adjustment for Body Mass index in Women from the Danish Diet, Cancer and Health Study. Abbreviations: HR, hazard ratio. WC, waist circumference. Lines are the hazard ratios (areas the 95%-confidence intervals) derived from Cox's proportional-hazard models where WC was included as restricted cubic splines (3 knots). Reference points are the mean WC. Years since the examination in 1999–02 was used as time axis. *Adjustments*: years between examinations, age in 1999–02, chronic diseases, smoking habits, Mediterranean diet score, energy intake, education, drinking pattern, sports activity, menopausal status. WC in 1993–97: Test of linearity *P* = 0.95 (linear). Test of effect *P*<0.0001 (with adjustment for covariates, but without adjustment for body mass index). WC in 1993–97: Test of linearity *P* = 0.45 (linear). Test of effect *P*<0.0001 (with adjustment for covariates and body mass index).

In women, the association between BMI in 1993–97 and diabetes was positive, but the association was weak after adjustment for WC (table 2, figure 4). The association between WC in 1993–97 and diabetes was positive (table 2, figure 5). A 5 cm larger WC was associated with a 31% (HR = 1.31 (1.23∶1.38) per 5 cm) higher risk of diabetes after adjusting for covariates and BMI.

The association between BMI in 1993–97 and diabetes was different in men and women after adjusting for covariates and WC (interaction, *P*<0.01). There was no significant sex difference between WC in 1993–97 and diabetes after adjusting for covariates and BMI (interaction, *P* = 0.34).

Associations between BMI and WC in 1999–02 and diabetes in men and women were fairly similar to those of BMI and WC in 1993–97 (Table 2 and figure S1).

### Changes in BMI and changes in WC

**Figure 6:**
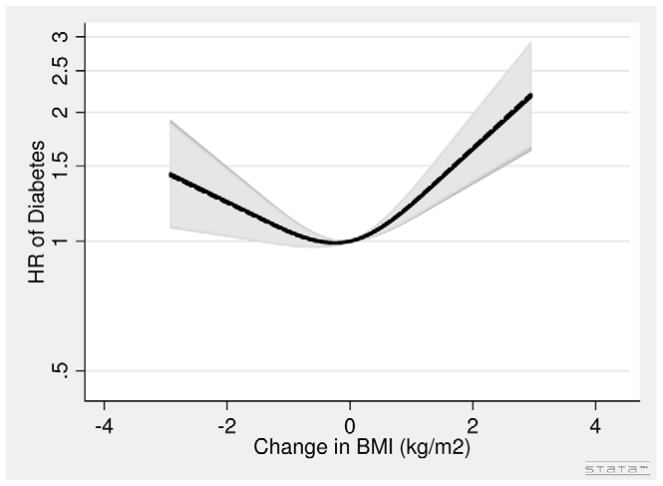
Hazard Ratios and 95% Confidence Intervals of Diabetes according to Changes in Body Mass Index without (dashed line) and with (solid line) adjustment for Changes in Waist Circumference in Men from the Danish Diet, Cancer and Health Study. Abbreviations: BMI, body mass index. DBMI, changes in body mass index. DWC, changes in waist circumference. HR, hazard ratio. WC, waist circumference. Lines are the hazard ratio (areas the 95%-confidence intervals) derived from Cox's proportional-hazard models where DBMI was included as restricted cubic splines (3 knots). The reference points are the mean DBMI. Years since the examination in 1999–02 was used as time axis. *Adjustments:* years between examinations, age in 1999–02, chronic diseases, smoking habits, Mediterranean diet score, energy intake, education, drinking pattern, sports activity. *Additional adjustments:* dotted line of DBMI (BMI in 1993–97) and solid line of DBMI (WC in 1993–97, BMI in 1993–97 and DWC). DBMI: Test of linearity *P*<0.0001 (nonlinear). Test of effect p<0.0001 (with adjustment for covariates, but without adjustment for DWC). DBMI: Test of linearity *P*<0.0001 (nonlinear). Test of effect *P*<0.0001 (with adjustment for covariates and DWC).

**Figure 7:**
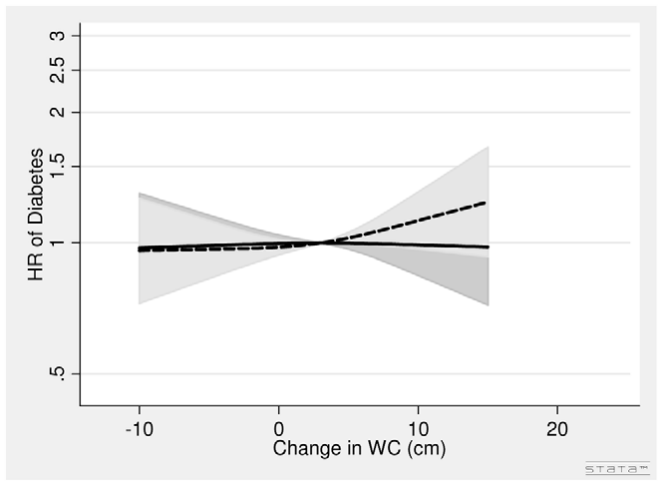
Hazard Ratios and 95% Confidence Intervals of Diabetes according to Changes in Waist Circumference (lower panel) without (dashed line) and with (solid line) adjustment for Changes in Body Mass Index in Men from the Danish Diet, Cancer and Health Study. Abbreviations: BMI, body mass index. DBMI, changes in body mass index. DWC, changes in waist circumference. HR, hazard ratio. WC, waist circumference. Lines are the hazard ratio (areas the 95%-confidence intervals) derived from Cox's proportional-hazard models where DWC was included as restricted cubic splines (3 knots). The reference points are the mean DWC. Years since the examination in 1999–02 was used as time axis. *Adjustments:* years between examinations, age in 1999–02, chronic diseases, smoking habits, Mediterranean diet score, energy intake, education, drinking pattern, sports activity. *Additional adjustments:* Dotted line of DWC (WC in 1993–97) and solid line of DWC (WC in 1993–97, BMI in 1993–97 and DBMI). DWC: Test of linearity *P* = 0.45 (linear). Test of effect *P* = 0.20 (with adjustment for covariates, but without adjustment for DBMI). DWC: Test of linearity *P* = 0.85 (linear). Test of effect *P* = 0.98 (with adjustment for covariates and DBMI).

In men, the association between DBMI and diabetes was J-shaped with the nadir of the curve at DBMI = 0 (figure 6). Thus, for men with loss of BMI (DBMI< = 0) one kg/m^2^ decrease in BMI was associated with a 14% (HR = 1.14 (1.03∶1.26)) higher risk of diabetes, whereas for men with gain in BMI (DBMI>0) one kg/m^2^ increase in BMI was associated with a 31% (HR = 1.31 (1.18∶1.45)) higher risk of diabetes after adjusting for covariates, BMI in 1993–97, WC in 1993–97 and DWC. The DWC was not associated with diabetes in men (figure 7, table 3). The HR was 1.00 (0.94, 1.07) per 5 cm change after adjusting for covariates BMI in 1993–97, WC in 1993–97 and DBMI (table 3).

**Table 3:** Hazard Ratios and 95% Confidence Intervals of Diabetes according to Changes in Waist Circumference and Changes in Body Mass Index in Men and Women from the Danish Diet, Cancer and Health Study.

	Crude	Adjusted	Adjusted+DWC	Adjusted+DBMI
Men	HR (95% CI)^c^	HR (95% CI)^c^^,^ d	HR (95% CI)^c^^,^ d ^,^ e	HR (95% CI)^c^^,^ d ^,^ e
DBMI (kg/m^2^)^f^	-	-	-	-
DWC (5 cm)	1.05 (0.99, 1.11)	1.05 (0.99. 1.12)	-	1.00 (0.94, 1.07)^b^
**Women**				
DBMI (kg/m^2^)	1.16 (1.11, 1.22)	1.16 (1.10, 1.22)	1.12 (1.06, 1.18)^a^	-
DWC (5 cm)	1.14 (1.09, 1.19)	1.14 (1.09, 1.19)	-	1.09 (1.04, 1.15)^b^

Abbreviations: CI, confidence interval. DBMI, changes in body mass index. DWC, changes in waist circumference. HR, hazard ratio.

a The association was different in men and women (interaction, *P*<0.05).

b The association was different in men and women (interaction, *P*<0.05).

c Adjusted for years between examinations, age in 1999–02, chronic diseases, BMI in 1993–97 (analyses of DBMI) or WC in 1993–97 (analyses of DWC).

d Adjusted for smoking habits, Mediterranean diet score, energy intake, education, drinking pattern, sports activity, menopausal status (in women only), BMI in 1993–97 (in analyses of DBMI) or WC in 1993–97 (in analyses of DWC).

e DWC and WC in 1993–97 added to analyses of DBMI and DBMI and BMI in 1993–97 added to analyses of DWC.

f Associations were accepted to be linear, except DBMI in men. Linear estimates for the association between DBMI and diabetes are therefore not shown in table 4, but the association is presented in figure 4. Furthermore, estimates for DBMI in groups with loss (DBMI< = 0) and gain in BMI (DBMI>0) in men are provided in the text.

**Figure 8:**
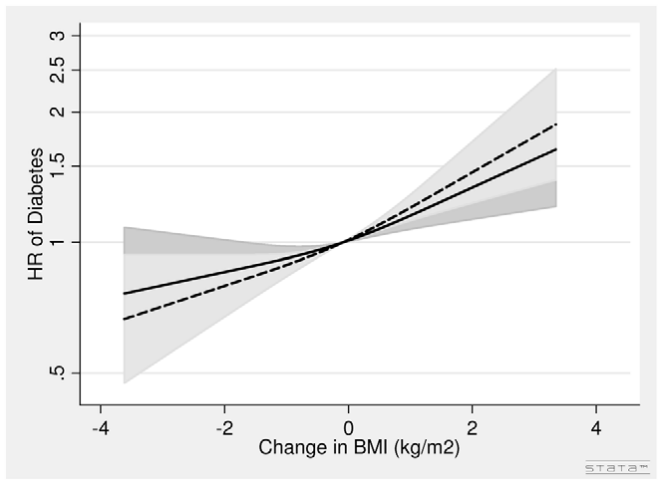
Hazard Ratios and 95% Confidence Intervals of Diabetes according to Changes in Body Mass Index without (dashed line) and with (solid line) adjustment for Changes in Waist Circumference in Women from the Danish Diet, Cancer and Health Study. Abbreviations: BMI, body mass index. DBMI, changes in body mass index. DWC, changes in waist circumference. HR, hazard ratio. WC, waist circumference. Lines are the hazard ratio (areas the 95%-confidence intervals) derived from Cox's proportional-hazard models where DBMI was included as restricted cubic splines (3 knots). The reference points are the mean DBMI. Years since the examination in 1999–02 was used as time axis. *Adjustments:* years between examinations, age in 1999–02, chronic diseases, smoking habits, Mediterranean diet score, energy intake, education, drinking pattern, sports activity, menopausal status. *Additional adjustments*: Dotted line of DBMI (BMI in 1993–97) and solid line of DBMI (WC in 1993–97, BMI in 1993–97 and DWC). DBMI: Test of linearity *P* = 0.39 (linear). Test of effect *P*<0.0001 (with adjustment for covariates, but without adjustment for DWC). DBMI: Test of linearity *P* = 0.40 (linear). Test of effect *P*<0.01 (with adjustment for covariates and DWC).

**Figure 9:**
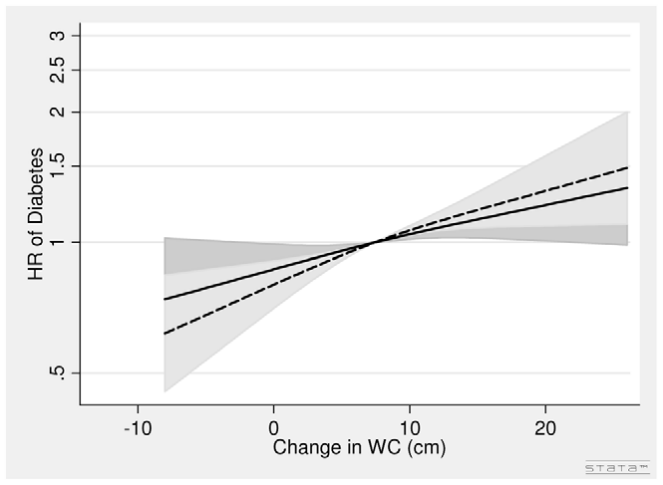
Hazard Ratios and 95% Confidence Intervals of Diabetes according to Changes in Waist Circumference (lower panel) without (dashed line) and with (solid line) adjustment for Changes in Body Mass Index in Women from the Danish Diet, Cancer and Health Study. Abbreviations: BMI, body mass index. DBMI, changes in body mass index. DWC, changes in waist circumference. HR, hazard ratio. WC, waist circumference. Lines are the hazard ratio (areas the 95%-confidence intervals) derived from Cox's proportional-hazard models where DWC was included as restricted cubic splines (3 knots). The reference points are the mean DWC. Years since the examination in 1999–02 was used as time axis. *Adjustments:* years between examinations, age in 1999–02, chronic diseases, smoking habits, Mediterranean diet score, energy intake, education, drinking pattern, sports activity, menopausal status. *Additional adjustments:* Dotted line of DWC (WC in 1993–97) and solid line of DWC (WC in 1993–97, BMI in 1993–97 and DBMI). DWC: Test of linearity *P* = 0.53 (linear). Test of effect *P*<0.0001 (with adjustment for covariates, but without adjustment for DBMI). DWC: Test of linearity *P* = 0.81 (linear). Test of effect *P*<0.05 (with adjustment for covariates and DBMI).

In women, the associations between DBMI, DWC and diabetes were positive (figure 8, 9, table 3). One unit (kg/m^2^) increase in BMI was associated with a 12% (HR = 1.12 (1.06∶1.18)) higher risk of diabetes after adjusting for covariates BMI in 1993–97, WC in 1993–97 and DWC. A 5 cm increase in WC was associated with a 9% (HR = 1.09 (1.04∶1.15)) higher risk of diabetes after adjusting for covariates, WC in 1993–97, BMI in 1993–97 and DBMI.

The association between DBMI and diabetes was significantly different in men and women after adjusting for covariates, BMI in 1993–97, WC in 1993–97 and DWC (interaction, *P* = 0.02), and so was the association between DWC and diabetes after adjusting for covariates, WC in 1993–97, BMI in 1993–97 and DBMI (interaction, *P* = 0.01).

### Subgroup analyses

The DWC was not associated with diabetes in men in the two strata of DBMI, whereas the association was positive in both strata in women (table 4). A 5 cm increase in WC was associated with a 7% and 11% higher risk of diabetes in women with loss and gain in BMI after adjusting for covariates, WC in 1993–97, BMI in 1993–97 and DBMI (Table 4).

**Table 4:** Hazard Ratios and 95% Confidence Intervals of Diabetes according to Changes in Waist Circumference in Strata of Changes in Body Mass Index in Men and Women from the Danish Diet, Cancer and Health Study.

	HR (95% CI)^a^	HR (95% CI)^a^	HR (95% CI)^a^	HR (95% CI)^a^
Men	Loss of BMI	Gain in BMI	Low WC in 1993–97	High WC in 1993–97
DWC (5 cm)	1.00 (0.91, 1.11)	1.00 (0.92, 1.10)	0.94 (0.82, 1.07)	1.03 (0.95, 1.11)
**Women**				
DWC (5 cm)	1.07 (0.99, 1.15)	1.11 (1.03, 1.19)	1.08 (0.98, 1.20)	1.09 (1.03, 1.16)

Abbreviations: BMI, body mass index. CI, confidence interval. DBMI, changes in body mass index. HR, hazard ratio. WC, waist circumference.

a Adjusted for years between examinations, age in 1999–02, chronic diseases, WC in 1993–97, BMI in 1993–97, DBMI, smoking habits, Mediterranean diet score, energy intake, education, drinking pattern, sports activity and in women also menopausal status.

All associations were accepted to be linear.

The DWC was not associated with diabetes in men in the two strata of WC in 1993–97, whereas the association between was positive in both strata in women (table 4). A 5 cm increase in WC was associated with 8% and 9% higher risk of diabetes in women with low and high WC after adjusting for covariates, WC in 1993–97, BMI in 1993–97 and DBMI (table 4).

The exclusion of individuals with chronic diseases and cases of diabetes occurring within the first years of follow-up had no notable influence on the associations between DWC and diabetes in men or between DWC, DBMI and diabetes in women. The higher risk of diabetes associated with loss of BMI in men (figure 6) was, however, attenuated. The DBMI was e.g. positively associated with diabetes after adjusting for covariates BMI in 1993–97, WC in 1993–97 and DWC when cases in the first five years of follow-up were excluded (HR = 1.33 (1.05, 1.68) per kg/m^2^ DBMI) (table 5).

**Table 5:** Hazard Ratios and 95% Confidence Intervals of Diabetes according to Changes in Waist Circumference (DWC) and Changes in Body Mass Index (DBMI) in Men and Women from the Danish Diet, Cancer and Health Study when Individuals with Chronic Diseases and Diabetes Cases occurring in the first years of follow-up are excluded.

Years of follow-up excluded	0 year	1 year	3 years	5 years
Men	HR (95% CI)^a^	HR (95% CI)^a^	HR (95% CI)^a^	HR (95% CI)^a^
DBMI (kg/m^2^)	1.09 (1.00, 1.17)^b^	1.09 (1.00, 1.19)^b^	1.10 (0.98, 1.23)^b^	1.33 (1.05, 1.68)
DWC (5 cm)	0.97 (0.89, 1.06)	0.96 (0.87, 1.05)	0.89 (0.79, 1.02)	0.99 (0.84, 1.17)
**Women**				
DBMI (kg/m^2^)	1.14 (1.07, 1.22)	1.15 (1.07, 1.24)	1.09 (0.98, 1.21)	1.05 (0.85, 1.30)
DWC (5 cm)	1.08 (1.01, 1.15)	1.08 (1.01, 1.16)	1.10 (1.00, 1.21)	1.12 (0.93, 1.38)

Abbreviations: CI, confidence interval. DBMI, changes in body mass index. DWC, changes in waist circumference. HR, hazard ratio.

a Adjusted for years between examinations, age in 1999–02, chronic diseases, WC in 1993–97, BMI in 1993–97, DWC (analyses of DBMI), DBMI (analyses of DWC), smoking habits, Mediterranean diet score, energy intake, education, drinking pattern, sports activity and in women also menopausal status.

b All associations were accepted to be linear, except^b^.

## Discussion

This prospective study of middle-aged men and women showed that WC at both recruitment and follow-up, as expected, was strongly and positively associated with the subsequent risk of diabetes also with adjustment for BMI. Changes in WC were, however, not associated with the risk of diabetes in men and only weakly positively associated with the risk of diabetes in women. These associations were not notably affected by adjustment for changes in BMI. Thus, the risk of diabetes was not modified by changes in WC as predicted from the association with WC made at a single point in time.

The strengths of the study are the large, well-characterized and ethnic homogeneous study population where information on changes in WC was available throughout a broad range of WC, BMI and BMI changes in both men and women. Selection bias is unlikely to have affected the results, as all study participants were followed after their second measurement of anthropometry until death or end of follow-up, and the number of participants lost due to death was low. The risk of information bias with respect to diagnosed diabetes was also low, as diabetes was assessed by record linkage to the Danish Diabetes Register independently of the collection of information regarding anthropometry.

The Diabetes Register [21], [22] includes persons on the basis on their contacts with the health care system and covers our entire cohort. The register is developed from a detailed study where limited misclassification of cases was found [22]. Up to 60% of individuals with diabetes are, however, unaware of their disease [27], and the actual number of cases may have been underestimated. Undiagnosed or sub-clinical diabetes may induce changes in anthropometry [26], which imply risks of bias due to reverse causation. We used a prospective study design, which reduces, but do not eliminate, the risk of reverse causality. A variety of other chronic diseases may also induce changes in anthropometry, and we adjusted for presence of diagnosed diseases [19]. The registries used to identify the diseased individuals are fairly complete and valid [20], but individuals with sub-clinical or psychiatric diseases were not identified. We can therefore not exclude the influence from undiagnosed diseases associated with both changes in anthropometry and diabetes. We conducted a series of analyses were individuals with diagnosed diseases and diabetes occurring in the first years of follow-up were excluded. The exclusions had no notable influence on the associations between changes in WC and diabetes, but the j-shaped association between changes in BMI and diabetes in men became linear indicating some reverse causality.

Residual confounding from other risk factors cannot be excluded, but we included a variety of covariates that could have confounding or modifying effects, but these had no major effects on the direction and strength of the associations.

Measurement problems may have had minimal impact on the baseline results, as trained technicians measured anthropometry in 1993–97. The anthropometric measures in 1999–2002 were self-reported. Strong, quantitatively consistent associations between diabetes and both baseline and follow-up anthropometric measures were observed. The different measurement methods of WC employed in 1993–97 (technicians) and 1999–02 (self-report) may have implications for the analyses of changes in WC. A validation study within this cohort [28] found that the mean change in WC was overestimated in women (2.1 cm) and slightly underestimated in men (0.8 cm), and that the difference was associated with BMI in men and with WC in women. It was, however, concluded that the two measures could be used together in analyses of changes in WC if the statistical models were adjusted for BMI and WC measured in 1993–97 [28]. Accordingly, in analyses of changes in WC we adjusted for BMI and WC in 1993–97. We also excluded individuals with extreme anthropometric measurements as misreporting may be most pronounced in these individuals. Perhaps more important, information on WC was collected years before the diabetes status, and it is thus unlikely that the over/underestimation of changes in WC is directly related to diabetes, which limits the risk of bias. Still we cannot exclude that measurement errors may have attenuated the results. However, we have previously shown that changes in WC is positively associated with mortality in the present cohort [29], and we therefore expect that the used measure of DWC would capture most of the effects on diabetes that exist in the data.

Fatness, and in particular abdominal fatness, is assumed to have an important role in the aetiology of diabetes [2]–[9]. This was also shown in our study, as WC was strongly associated with the risk of diabetes also after adjustment for BMI. Changes in WC were, however, not associated with the risk of diabetes in men and only weakly positively associated with the risk of diabetes in women. These findings may thus suggest that it is not possible to predict the risk of diabetes associated with changes in WC from the risk associated with differences in WC measured at one point in time. It seems likely that the association with WC at one point in time reflects lifelong exposure, whereas the risk associated with changes in WC reflects the individual possibility to modulate such lifelong risk during a short five-year period.

Changes in WC were positively associated with diabetes in women, but not in men. One explanation for this sex difference could be the age range of our participants. Our participants were 50–64 years at baseline, and it may hence be suspected that the men already had redistributed their fat mass to the abdominal fat depots [30], [31], and therefore were too old to influence their risk of diabetes by changes in WC. In contrast, women may redistribute their fat towards the abdominal depots in the years around menopause [30], [31]. Our women could therefore be more susceptible to diabetes as a consequence of changes in WC. In the younger CARDIA population, changes in WC were positively associated with the incidence of diabetes in both men and women, although estimates were rather weak and not adjusted for overall weight changes [12]. Changes in WC were also associated with diabetes in men aged 40–75 years in the Health Professionals Follow-up Study [13], but only the highest quintile of WC gain (≥14.6 cm) was associated with a higher risk of diabetes and the association was weak after adjustment for overall weight change [13], which correspond to our results in men. None of these studies provided results pertaining only to women, but in accordance with our findings, it has been proposed that accumulation of intra-abdominal fat mass in women, who typically carry lower amounts of intra-abdominal fat than men, reflect a more insidious or advanced state of metabolic deterioration [8], [9]. Moreover, triacylglyceride may in itself be an inert compound when deposited as intracellular droplets [32], [33]. Metabolic dysfunctions may therefore first occur when the ability to expand the fat tissue and store the triacylglyceride is exceeded. Such storage capacity may depend on various factors e.g. sex and age, and waist gain may be seen as a stepwise process for exceeding such ability.

Adjustment for changes in BMI had no major influence on the association between changes in WC and diabetes possibly due to the modest correlation between changes in WC and changes in BMI [13]. The adjustment does not only reduce confounding, but does also introduce a substitution aspect in the interpretation of the results. The higher risk of diabetes associated with an increase in WC in women may e.g. be explained by gain in harmful abdominal fat or by loss of beneficial peripheral fat or lean body mass that may accompany the WC gain as changes in BMI are fixed. These effects cannot be directly separated from the results, but underscore that redistribution of fat mass towards the abdominal region is a risk factor for diabetes in middle-aged women.

In conclusion, this study confirmed that WC was strongly and positively associated with the risk of diabetes in middle-aged men and women. Changes in WC were, however, not associated with the risk of diabetes in men and only weakly positively associated with the risk of diabetes in women. These associations were not notably affected by adjustment for changes in BMI. According to these findings it is not possible to predict the risk of diabetes following changes in WC from studies where WC is only measured at one point in time. A reduction in WC may hence be a weak or insufficient target for prevention of diabetes in middle-aged men and women.

## Supporting Information

Figure S1 **Hazard Ratios and 95% Confidence Intervals of Diabetes according to Body Mass Index and Waist Circumference in 1999–02 without mutual adjustment (dashed line) and with mutual adjustment (solid line) in Men (upper panel) and Women (lower panel) from the Danish Diet, Cancer and Health Study.**
(PDF)
