# Supplementary material for: Changes in Waist Circumference and the Incidence of Diabetes in Middle-Aged Men and Women
Source: PLoS One. 2011 Aug 4;6(8):e23104. doi: 10.1371/journal.pone.0023104 (PMC3150401; doi:10.1371/journal.pone.0023104)
Supplement: Figure S1 — Hazard Ratios and 95% Confidence Intervals of Diabetes according to Body Mass Index and Waist Circumference in 1999–02 without mutual adjustment (dashed line) and with mutual adjustment (solid line) in Men (upper panel) and Women (lower panel) from the Danish Diet, Cancer and Health Study. (PDF) [file pone.0023104.s001.pdf]

Legend figure S1

Abbreviations: BMI, body mass index. HR, hazard ratio. WC, waist circumference

Lines are the hazard ratios (areas the 95%-confidence intervals) derived from Cox's proportional-hazard models where WC and BMI were included as restricted cubic splines (3 knots). Reference points are the respective means of WC and BMI. Years since the examination in 1999-02 was used as time axis.

Adjustments: years between examinations, age in 1999-02, chronic diseases, smoking habits, Mediterranean diet score, energy intake, education, drinking pattern, sports activity and menopausal status (women only).

Men

BMI in 1999-02: Test of linearity P= 0.62 (linear). Test of effect P< 0.0001 (with adjustment for covariates, but without adjustment for WC)

BMI in 1999-02: Test of linearity P= 0.77 (linear). Test of effect P< 0.0001 (with adjustment for covariates and WC)

WC in 1999-02: Test of linearity $P=0.89$ (linear). Test of effect P< 0.0001 (with adjustment for covariates, but without adjustment for BMI)

WC in 1999-02: Test of linearity $P=0.86$ (linear). Test of effect P< 0.01 (with adjustment for covariates and BMI)

Women

BMI in 1999-02: Test of linearity P= 0.04 (nonlinear). Test of effect P< 0.0001 (with adjustment for covariates, but without adjustment for WC)

BMI in 1999-02: Test of linearity P= 0.17 (linear). Test of effect P< 0.0001 (with adjustment for covariates and WC)

WC in 1999-02: Test of linearity P= 0.81 (linear). Test of effect P< 0.0001 (with adjustment for covariates, but without adjustment for BMI)

WC in 1999-02: Test of linearity P= 0.79 (linear). Test of effect P< 0.0001 (with adjustment for covariates and BMI)

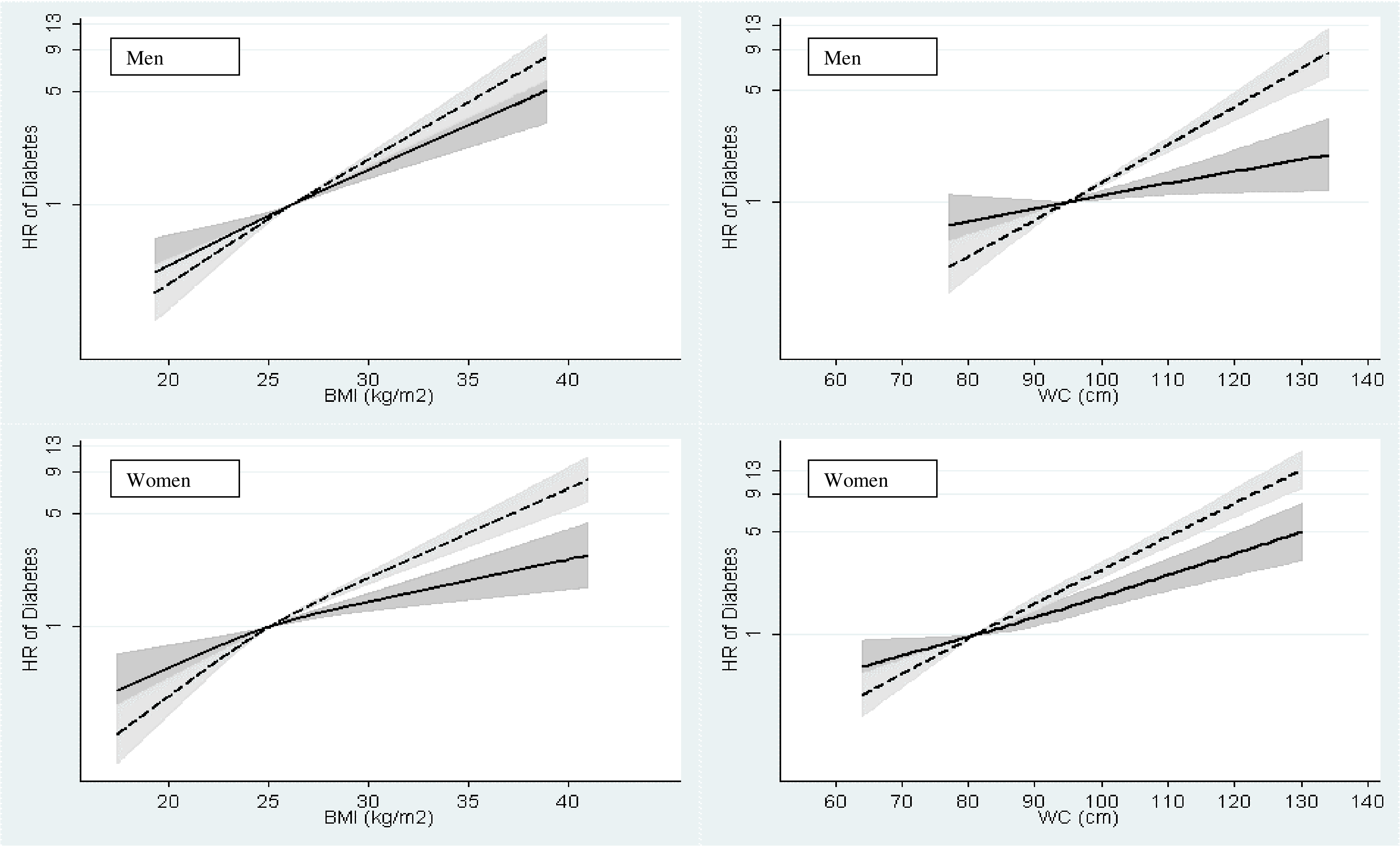
Men
HR of Diabetes
1
5
9 13
20
25
30
35
40
BMI (kg/m2)
Men
HR of Diabetes
60
70
80
90
100
110
120
130
140
WC (cm)
Women
HR of Diabetes
BMI (kg/m2)
Women
HR of Diabetes
WC (cm)
